# Characterization of two complete chloroplast genomes of *Lindera megaphylla* (Lauraceae)

**DOI:** 10.1080/23802359.2019.1660275

**Published:** 2019-09-09

**Authors:** Kai Jiang, Zheng-Wei Wang, Wei-Chang Huang, Yong-Hong Hu

**Affiliations:** a Shanghai Chenshan Plant Science Research Center, Chinese Academy of Sciences, Chenshan Botanical Garden, Shanghai, China;; b Shanghai Key Laboratory of Plant Functional Genomics and Resources, Shanghai Chenshan Botanical Garden, Shanghai, China;; c School of Ecological and Environmental Sciences, Shanghai Key Lab of Urban Ecological Processes and Eco-Restoration, East China Normal University, Shanghai, China

**Keywords:** *Lindera megaphylla*, Illumina sequence, chloroplast genome, phylogenetic position

## Abstract

*Lindera megaphylla* (Lauraceae) is an ecologically important and dominant evergreen broad-leaf tree species in the warm-temperate and subtropical zone of China. In this study, we sequenced and assembled two complete genomes of *L. megaphylla* (LM01 and LM02) based on the next-generation sequencing data. The two complete chloroplasts of *L. megaphylla* are 152,741 bp in length, including two same length inverted repeats of 20,067 and 20,068 bp, a small single copy of 18,882 and 18,914 bp and a large single copy of 93,726 and 93,691 bp for LM01 and LM02 respectively. They all contain 84 protein-coding, 36 tRNA and eight rRNA genes. The phylogenetic analysis based on chloroplast genomes indicates that *L. megaphylla* is closely related to another *Lindera* and *Litsea* species.

*Lindera* (Lauraceae) is a genus of flowering plants including nearly 100 species distributed in tropical and subtropical Asia and North American (Cao et al. 2016). About 40 species of *Lindera* distributed widely in China indicated high species diversity in this region. *Lindera megaphylla* Hemsl. is an ecologically important and dominant evergreen broad-leaf tree species in the warm-temperate and subtropical zone of China. In this study, we reported two cp genomes of *L. megaphylla* from a different location. The results will provide sufficient information for species identification and phylogenetic studies of *Lindera*.

We collected fresh leaves of *L. megaphylla* from two living plants located in Zhejiang province (LM01: 28.81°N 120.74°E; LM02: 28.35°N 121.34°E) of China and the specimen stored at the Shanghai Chenshan Botanical Garden Herbarium (CS-JK20150201 and CS-JK20150202). About 50 g of the fresh leaves were obtained and cleaned with 75% alcohol and distilled water, and then these materials were restored in 4 °C refrigerator. The total chloroplast genomic DNA was extracted according to the high salt methods modified by Shi et al. (2012).

Using the Illumina Miseq system, two *L. megaphylla* individuals were sequenced to produce 1,585,773 and 1,624,370 paired-end clean reads after screening out low-quality data. These paired-end reads were assembled using MITObim v1.8 (Hahn et al. 2013). We used a Dual Organeller GenoMe Annotator (DOGMA) software (Wyman et al. 2004) to annotate the chloroplast genome initially. These annotations were manually corrected for the start and stop codons and intro/exon boundaries by comparison to homologous genes in *L. megaphylla* cp genome (GenBank accession no. NC_035953). These tRNA genes were also verified with tRNAscan-SE (Lowe and Eddy 1997).

The complete cp genomes sequences of *L. megaphylla* (GenBank: MK937812) with a length of 152,741bp, was the same with another individual (GenBank: MK937813). The two cp genomes possessed a typical quadripartite structure, consisting of a paired of IRs, separated by the LSC and SSC regions. The two individuals had the same GC content (33.9%). However, the GC contents of the LSC and SSC regions (33.9 and 38.0%) were lower than those of the IR regions (44.4%). Total of 128 predicted functional genes were found through the annotation of DOGMA. Each cp genomes contained 84 protein-coding, 36 tRNA and eight rRNA genes. One hundred and eleven genes were unique, including 80 protein-coding, 27 tRNA genes, and four rRNA genes. LSC region comprised 66 protein-coding genes and 20 tRNA genes, whereas 12 protein-coding genes and one tRNA gene were found in the SSC region. Five protein-coding and six tRNA genes were repeated in the IR regions.

To determine the systematic position of the two new *L. megaphylla* cp genome, we constructed a molecular phylogenetic tree using the whole chloroplast genome of 34 angiosperms. We reconstructed the phylogenetic relationships using Bayesian inference (BI) methods in MrBayes version 3.1.2 (Huelsenbeck and Ronquist 2005) using GTR + I+G model. *Magnolia officinalis* was set as outgroup among these 34 taxa. Our results showed that our samples LM01 and LM02 were closed to another *L. megaphylla* (Figure 1).

**Figure 1.**
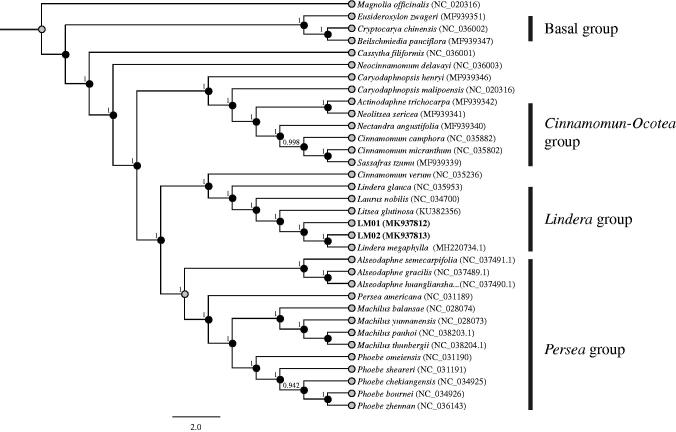
Phylogenetic relationships of 33 Lauraceae species and one outgroup species based on the whole chloroplast genomes. Numbers at the nodes represent Bayesian inference posterior probabilities (BI-PP).

## Geolocation information

Zhejiang Province, China (LM01: 28.81°N 120.74°E; LM02: 28.35°N 121.34°E).
